# Secondary Neoplasm in Survivors of Childhood Hematological Malignancies—Systematic Review

**DOI:** 10.3390/children13020205

**Published:** 2026-01-31

**Authors:** Ioana-Alexandra Horneț, Andreea Bianca Stoica, Dora Mihaela Cîmpian, Lucian Puşcaşiu

**Affiliations:** 1 Doctoral School of Medicine, George Emil Palade University of Medicine, Pharmacy, Science and Technology of Târgu Mureș, Gheorghe Marinescu Street No. 38, 540136 Târgu Mureș, Romania; alexandra.hornet@gmail.com (I.-A.H.); puscasiu@gmail.com (L.P.); 2 Department of Pediatrics 1, George Emil Palade University of Medicine, Pharmacy, Science and Technology of Târgu Mureș, Gheorghe Marinescu Street No 38, 540136 Târgu Mureș, Romania; 3 Department of Ethics and Social Sciences, George Emil Palade University of Medicine, Pharmacy, Science and Technology of Târgu Mureș, 540139 Târgu Mureş, Romania; dora.cimpian@umfst.ro; 4 Department of Obstetrics and Gynecology 1, George Emil Palade University of Medicine, Pharmacy, Science and Technology of Târgu Mureș, Gheorghe Marinescu Street No. 38, 540136 Târgu Mureș, Romania

**Keywords:** childhood cancer survivors, hematologic malignancies, secondary malignant neoplasms, Hodgkin lymphoma, acute lymphoblastic leukemia, hematopoietic stem cell transplantation, radiotherapy, survivorship, PRISMA priorities for research and policy

## Abstract

Background: Childhood cancers account for approximately 1–2% of all malignancies worldwide, with hematologic cancers representing about 35–40% of pediatric cases. Improved survival has brought increased recognition of both acute and long-term therapy-related complications, including secondary malignant neoplasms (SMNs). Survivors of pediatric hematologic malignancies face a lifelong risk of secondary malignant neoplasms (SMNs), which remain among the most severe late effects of therapy. Methods: We conducted a PRISMA 2020–aligned systematic review of cohort and registry studies evaluating SMNs after childhood hematologic cancers. Databases searched included PubMed, Embase, Web of Science, Scopus, and Cochrane Library. Two reviewers independently screened studies, extracted data, and assessed risk of bias using the Newcastle–Ottawa Scale; disagreements were resolved by a third reviewer. Results: Forty-three studies (>70,000 survivors, median follow-up 5–30+ years) were included. Standardized incidence ratios (SIRs) for secondary malignant neoplasms compared to the general population ranged from 2.0 to 6.0, with absolute excess risks (AERs) of approximately 10–40 per 10,000 person-years. Therapy-related acute myeloid leukemia occurred within 5–10 years, while solid secondary malignant neoplasms (breast, thyroid, central nervous system, sarcomas) emerged after 10–25 years. The highest risks for developing secondary malignant neoplasms were observed among female survivors of Hodgkin lymphoma treated with chest and neck radiotherapy, particularly during adolescence, and among hematopoietic stem cell transplant recipients exposed to total body irradiation or chronic graft-versus-host disease. Conclusions: SMNs are predictable late effects requiring lifelong, exposure-anchored surveillance. Precision survivorship—integrating treatment exposures, transplant conditioning, and genetic predisposition—should guide future screening strategies.

## 1. Introduction

Pediatric cancers are among the leading causes of disease-related death in children and adolescents, despite their relatively low incidence compared to adult cancers. Recent analyses indicate a steadily rising global incidence, with rates ranging from 178–218 cases per million children and adolescents. Leukemias, lymphomas, and central nervous system (CNS) tumors are the most commonly diagnosed malignancies, particularly in boys and in younger age groups [1,2,3].

Outcomes for pediatric cancer have improved substantially in high-income countries due to advances in risk-adapted chemotherapy, radiotherapy, hematopoietic stem cell transplantation, and comprehensive supportive care. Five-year survival rates now exceed 80% in resource-rich environments, yet disparities in access to care and socioeconomic status remain prominent predictors of mortality, with significantly lower survival rates in low- and middle-income countries and among socioeconomically disadvantaged populations [4,5,6].

While survival rates have increased, the success of therapy is accompanied by a broad range of treatment-related complications. These adverse effects are generally categorized as acute (occurring during or shortly after therapy), chronic complications that persist or progress long-term after treatment, and late-onset complications, which emerge after a prolonged latency period, often months to decades following completion of therapy. Acute complications include infections, neutropenia, mucositis, and metabolic disturbances. Chronic complications encompass conditions such as cardiotoxicity, endocrine and fertility disorders, pulmonary dysfunction, and neurocognitive impairment. In contrast, late-onset complications—most notably secondary malignant neoplasms—develop after extended latency and represent some of the most severe long-term consequences of childhood cancer therapy. Studies indicate that up to 60–90% of childhood cancer survivors develop at least one chronic health condition, and up to 20–80% experience severe or life-threatening complications [7].

With increasing survival, long-term sequelae of cancer therapy have become more apparent. Survivors of childhood hematologic malignancies face a heightened risk of late complications, notably SMNs, which are histologically distinct cancers developing after primary therapy, excluding recurrences or metastases. The incidence and latency of SMNs are influenced by the type of primary cancer, treatment modalities such as radiation and specific chemotherapeutic agents, and underlying genetic predispositions. SMNs now represent one of the most severe late effects, accounting for substantial morbidity and mortality among long-term survivors [8,9,10].

This systematic review synthesizes cohort and registry data reporting SMNs in survivors of pediatric hematologic malignancies, aiming to quantify incidence rates, SIRs and AERs, to characterize SMN types and latency, identify treatment- and host-related risk factors, and evaluate temporal trends across treatment eras.

## 2. Materials and Methods

### 2.1. Search Strategy and Selection Criteria

This systematic review was conducted according to PRISMA 2020 guidelines. The objective was to narratively synthesize the available evidence on SMNs in survivors of pediatric hematologic malignancies, with a focus on incidence, latency, and risk factors related to treatment and host characteristics. We searched PubMed/MEDLINE, Embase, Web of Science, Scopus, and Cochrane Library for studies published between 2010 and 2024. Additional sources included registry reports and published literature. The search combined MeSH and key words such as “childhood cancer survivors”, “hematologic malignancies”, “HSCT”, and “total body irradiation”. References from relevant reviews and included articles were also screened manually.

### 2.2. Study Selection and Characteristics

Out of 43 studies initially screened, more than 30 met inclusion criteria, including both classical long-term survivor cohorts and recent publications. Collectively, these encompassed over 70,000 survivors from North America, Europe, and Asia, with median follow-up ranging from 5 to more than 30 years. Cohorts included the Childhood Cancer Survivor Study (CCSS, US/Canada), the British Childhood Cancer Survivor Study (BCCSS), the SEER database, several European national registries, as well as newer Asian population-based studies such as the Taiwan Registry (2022).

Inclusion criteria comprised papers containing data on survivors diagnosed with a hematologic malignancy before the age of 18 years; diagnosis of hematological cancers (the most commonly encountered being acute lymphoblastic leukemia [ALL], acute myeloid leukemia [AML], Hodgkin lymphoma [HL], and non-Hodgkin lymphoma [NHL]); survival of at least 5 years after initial diagnosis; publications that were prospective or retrospective cohort studies or registry studies; and studies reporting the incidence of SMNs, their latency, and/or providing SIR, hazard ratios (HR), AER, or cumulative incidence estimates. Exclusion criteria were papers that represented: case reports, case series without denominators, guidelines, narrative reviews, consensus statements, non-hematologic cancers, cohorts that assessed patients older than 18 years of age, studies with insufficient follow-up or unclear SMN definitions. Two reviewers independently screened studies. Two reviewers independently screened titles, abstracts, and full texts. Divergences were adjudicated by a third reviewer. Data extraction included cohort characteristics, follow-up, SMN counts, cumulative incidence, SIRs, AERs, and risk factors. Risk of bias was assessed using the Newcastle–Ottawa Scale.

PRISMA 2020 was applied in our paper selection for this review that was used to prompt a narrative synthesis of the subject discussed in our paper.

This review was conducted in accordance with the Preferred Reporting Items for Systematic Reviews and Meta-Analyses (PRISMA) 2020 statement (see Supplementary Material Table S1). The study was registered on the Open Science Framework (OSF) (registration DOI: https://doi.org/10.17605/OSF.IO/M97N4).

The search in the data bases identified a total number of 2034 articles. Before the screening 400 papers were eliminated, either because they were duplicates, or were rendered ineligible by automation tools and other 18 for different reasons (e.g. Lack of access to full text). After that, 1452 articles were screened based on title and abstract, out of which 400 were excluded based on relevancy to the topic.

**Figure 1:**
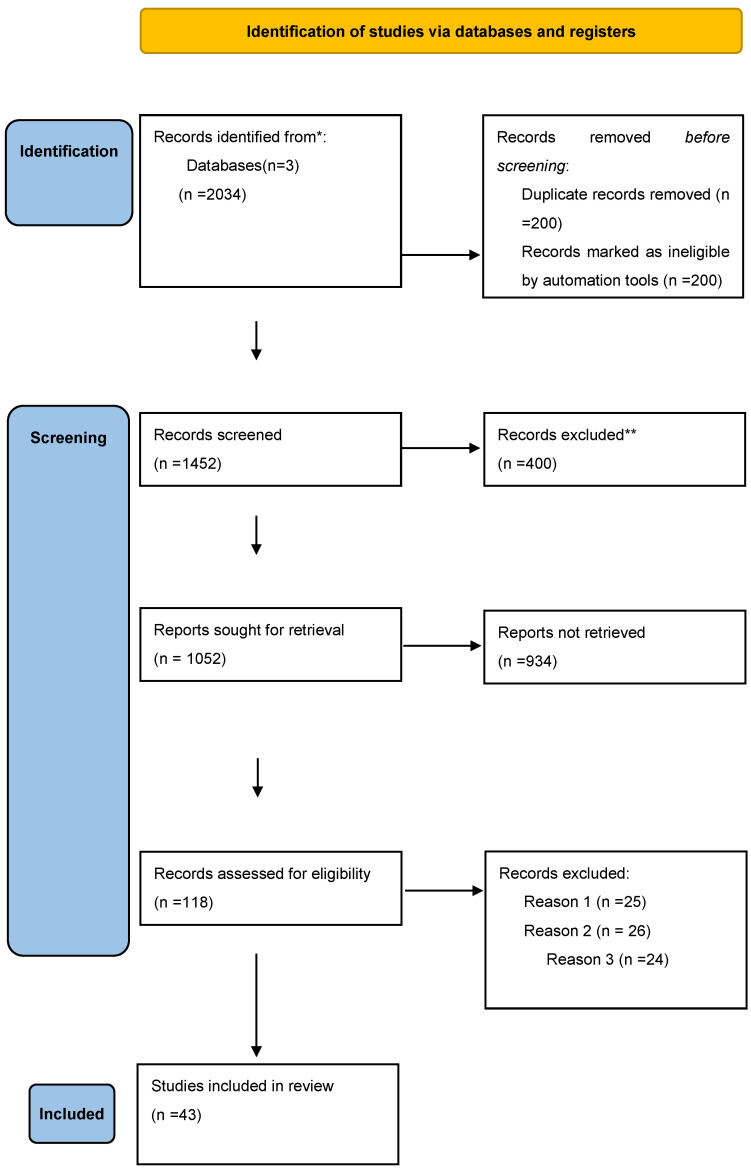
PRISMA 2020 flow diagram for new systematic reviews which included searches of databases and registers only. * The number of records identified from Google Scholar, PubMed and Cochrane databases. ** Records excluded by a human. Reason 1 Records excluded publications with insufficient follow-up period. Reason 2 Records excluded because they contained adult cohorts only. Reason 3 Records excluded based on unclear definition of SMNs.

Out of the remaining 1052 articles, we sought retrieval and to obtain full text access, unfortunately 934 were not retrievable (lack of access, retracted or unavailable papers) Finally, 118 full text articles were assessed for eligibility. Out of them 75 were excluded based on the following ciriteria: insufficient follow up period (25), adults only cohorts (26), unclear definiton of SMN (24). The selection process is summarized in Figure 1 [11].

### 2.3. Data Extraction and Quality Assessment

Data were extracted in duplicate using a standardized form. Extracted variables included author, year, country/registry, sample size, follow-up duration, number of SMNs, cumulative incidence, SIR (95% CI), AER, and HRs for risk factors.

Risk of bias was assessed with the Newcastle–Ottawa Scale (NOS), evaluating selection, comparability, and outcome domains.

The methodological quality of the included studies was assessed using the Newcastle–Ottawa Scale (NOS). According to this scale, up to 4 stars can be assigned for Selection, 2 for Comparability, and 3 for Outcome/Follow-up, with higher scores indicating better methodological quality.

**Figure 2:**
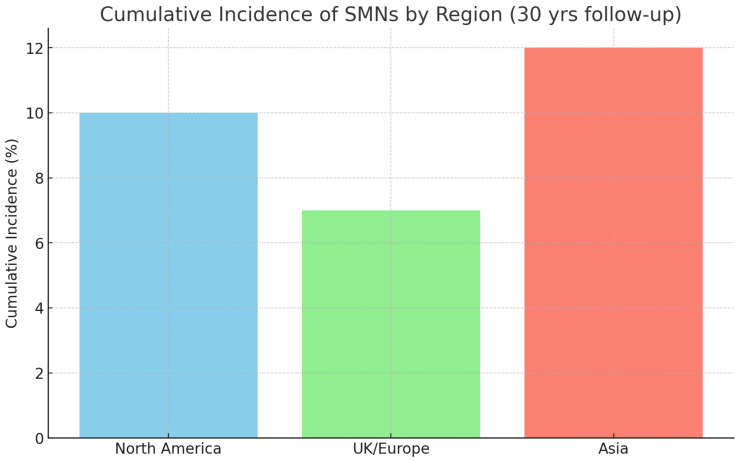
Regional variation in SMN incidence.

**Figure 3:**
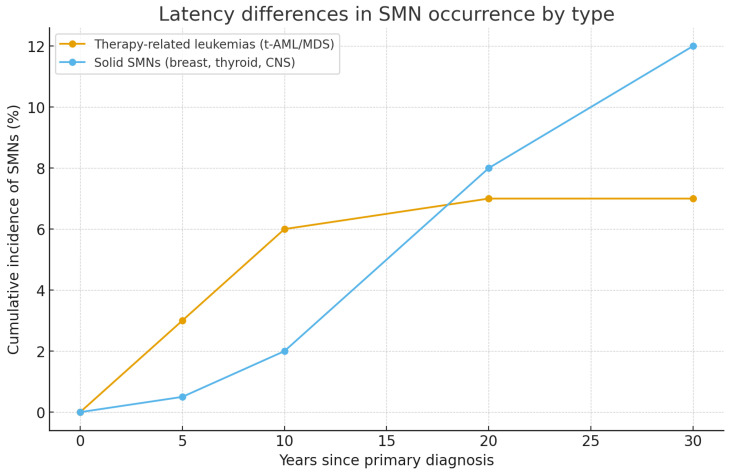
Latency differences in SMN occurrence by type.

**Figure 4:**
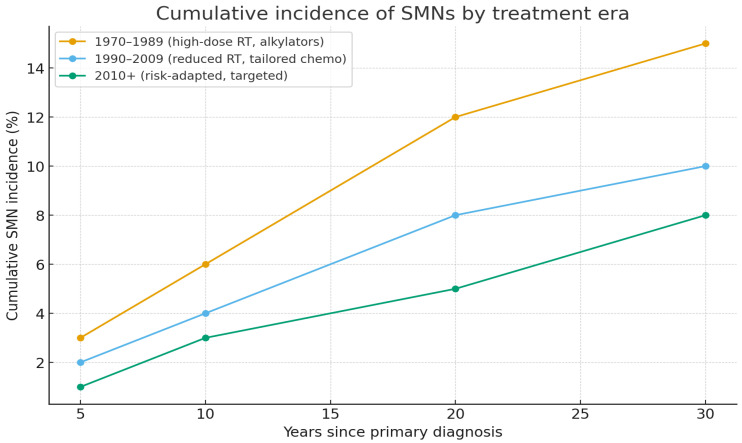
Cumulative incidence of SMNs by treatment era.

Owing to heterogeneity in design, treatment exposures, and reported outcomes, meta-analysis was not feasible. Due to substantial clinical, methodological, and statistical heterogeneity across included studies, a quantitative meta-analysis was not performed. Specifically, cohorts differed markedly in primary diagnoses (ALL, AML, HL, NHL), treatment eras, radiotherapy fields and doses, chemotherapy regimens, and use of hematopoietic stem cell transplantation. In addition, outcome definitions varied across studies, with heterogeneous reporting of SIRs, AERs, cumulative incidence estimates, and hazard ratios, often calculated over different follow-up intervals. Given this variability, pooling estimates would have risked generating misleading summary measures. Therefore, results were synthesized using a structured narrative approach, consistent with PRISMA 2020 recommendations for reviews with high heterogeneity. Instead, results were synthesized descriptively, supported by Table 1, Table 2, Table 3, Table 4 and Table 5 and Figure 1, Figure 2, Figure 3 and Figure 4.

The characteristics of the 43 studies included in this systematic review are summarized in Table 2, detailing cohort size, region, primary diagnosis, follow-up, main SMNs reported, and effect measures.

‘Multinational’ refers to cohorts including participants from multiple countries within the same geographic region (e.g., Western Europe, North America), reflecting shared clinical protocols and registry systems rather than unrelated nations.

Together, Table 1 and Table 2 provide a transparent overview of the characteristics and methodological quality of the included studies. Based on this evidence base, the following Results section summarizes the incidence, latency, and risk factors for secondary malignant neoplasms in survivors of pediatric hematologic malignancies.

## 3. Results

### 3.1. Incidence and Risk Estimates

Across studies, SIR for any SMN ranged from 2.0 to 6.0 compared with the general population. AER varied between 10 and 40 SMNs per 10,000 person-years, depending on primary cancer type, treatment exposure, and geographic region. Recent evidence confirms that the cumulative incidence of SMNs can reach or exceed 15% at 30 years post-diagnosis in selected cohorts, particularly among survivors of Hodgkin lymphoma treated with chest irradiation, as well as among Asian populations with elevated risks of thyroid carcinoma (predominantly papillary carcinoma) and primary liver malignancies, mainly hepatocellular carcinoma [14]. Survivors who underwent hematopoietic stem cell transplantation (HSCT) also exhibited persistently elevated risks of solid SMNs, especially in the presence of chronic graft-versus-host disease (GVHD) and total body irradiation (TBI)–based conditioning regimens [14].

Marked regional differences were observed. North American cohorts reported higher incidences of breast carcinoma (predominantly invasive ductal carcinoma) and thyroid carcinoma, European cohorts showed relatively higher proportions of CNS tumors and bone or soft tissue sarcomas, whereas Asian registries highlighted increased risks of thyroid carcinoma and liver malignancies. These findings suggest the influence of regional treatment practices, genetic susceptibility, and environmental factors. The results are summarized in Table 3 and illustrated in Figure 2.

### 3.2. Spectrum of SMNs

The spectrum of SMNs among survivors of pediatric hematologic malignancies is heterogeneous and shaped by both treatment-related and host-related factors. Therapy-related myeloid neoplasms—specifically therapy-related acute myeloid leukemia (t-AML) and therapy-related myelodysplastic syndrome (t-MDS)—represent the earliest occurring SMNs, with a typical latency of 5–10 years following exposure to alkylating agents, epipodophyllotoxins, or intensive HSCT conditioning regimens [15,16]. Importantly, t-MDS is a clonal bone marrow disorder that may progress to acute myeloid leukemia but is not itself leukemia.

Radiation-associated solid tumors, including CNS tumors, thyroid carcinoma (mainly papillary and follicular subtypes), and breast carcinoma (predominantly invasive ductal carcinoma), generally occur after longer latency periods of 10–25 years [14,17]. Breast cancer risk is particularly elevated in female Hodgkin lymphoma survivors treated with chest irradiation at a young age, with magnitudes comparable to those observed in carriers of *BRCA1* or *BRCA2* pathogenic variants [18].

Regional studies highlight important differences in the SMN spectrum. Data from the Taiwan Registry (2022) demonstrated disproportionately high rates of thyroid and liver cancers in Asian survivors compared with Western cohorts, reflecting both treatment differences and genetic susceptibility [19].

HSCT survivors remain a particularly high-risk group. Solid tumors of the oral cavity, skin, thyroid, and liver have been repeatedly associated with chronic GVHD and immunosuppressive therapies. A 2024 report emphasized persistent long-term risk, especially after total body irradiation (TBI)-based conditioning [15,19].

Genetic predisposition also contributes significantly to the diversity of SMNs. Germline *TP53* mutations (Li-Fraumeni syndrome) markedly increase susceptibility to both therapy-related leukemias and radiation-associated solid tumors, while variants of genes involved in DNA repair or telomere maintenance modulate risks across different cancer types [16].

Overall, the distribution of SMNs reflects the complex interplay between therapy, host susceptibility, and geographic context. While hematologic SMNs tend to occur early, solid tumors—including breast, thyroid, liver, and CNS cancers—dominate in long-term follow-up, often decades after the initial cancer diagnosis. Latency patterns varied markedly by type of SMN. Therapy-related leukemias (t-AML/MDS) peaked within the first decade after treatment, whereas solid tumors such as breast, thyroid, and CNS cancers accumulated progressively over the following decades. These trends complement the data shown in Table 4 and are depicted in Figure 3.

Line chart showing latency differences between therapy-related leukemias (early peak, plateau after 10 years) and solid SMNs (delayed onset, progressive rise up to 30 years).

Hematologic primaries such as ALL more frequently lead to secondary leukemias, while HL and NHL survivors show a predominance of solid SMNs, particularly breast and thyroid cancers.

Survivors who underwent HSCT presented distinct risk profiles, particularly for oral cavity, thyroid, skin, and liver cancers. As shown in Figure 3, these malignancies were strongly associated with conditioning regimens including total body irradiation and chronic graft-versus-host disease.

HSCT survivors presented distinct risk profiles, particularly for thyroid, oral cavity, skin, and liver cancers.

### 3.3. Risk Factors

Risk factors for SMN development can be broadly categorized as therapy-related or host-related. Therapy-related factors include radiotherapy dose and field size, exposure to alkylating agents, epipodophyllotoxins, anthracyclines, and HSCT. Survivors treated with high-dose radiotherapy—particularly chest or cranial irradiation—continue to exhibit the highest cumulative risks, as confirmed in both classical cohorts and recent updates [18].

Host-related factors include female sex, younger age at cancer diagnosis, and inherited cancer predisposition. Female survivors of Hodgkin lymphoma treated with chest irradiation before 20 years of age experience breast cancer risks comparable to those of BRCA mutation carriers [18]. Germline mutations in *TP53* and other cancer predisposition genes substantially increase susceptibility to both therapy-related myeloid neoplasms and radiation-associated solid tumors, while low-penetrance variants in DNA repair and telomere maintenance genes further modulate individual risk [21,44].

Treatment era is also a determinant of SMN risk. The incidence of SMNs is lower in survivors treated in the 2000s–2010s compared with those treated in the 1970s–1980s, reflecting the reduction in radiotherapy doses and the adoption of risk-adapted chemotherapy protocols [15]. Nonetheless, HSCT survivors, particularly those exposed to total body irradiation (TBI) and chronic GVHD, remain at elevated risk for both hematologic and solid SMNs [18]. Differences across treatment eras are summarized in Table 5.

Marked differences emerged across treatment eras. Survivors treated before 1990 had cumulative SMN risks exceeding 14% at 25–30 years, while those treated between 1990–2009 showed reduced risks of 6–10%, and post-2010 cohorts had risks below 10%, though longer follow-up is required. These differences are detailed in Table 5 and visualized in Figure 4.

While survivors treated before 1990 carried SMN risks exceeding 14%, cohorts treated after 2000 show substantially reduced risks, rarely above 10%. Nevertheless, the full impact of modern protocols will only become evident with longer follow-up.

Recent studies have refined risk stratification for SMNs. Large cohort analyses confirmed extremely high risks of breast and thyroid carcinoma among female Hodgkin lymphoma survivors treated with chest irradiation before 20 years of age, with risk magnitudes comparable to hereditary breast cancer syndromes [21]. Asian registry data highlighted a predominance of papillary thyroid carcinoma and hepatocellular carcinoma [22]. Among HSCT recipients, elevated incidences of oral cavity, thyroid, and liver cancers were strongly associated with chronic GVHD and intensive conditioning regimens, particularly TBI [14]. Finally, studies focusing on genetic susceptibility reaffirmed germline *TP53* mutations as strong predictors of multiple SMNs across diverse cancer types [16].

These recent studies illustrate both the persistence of high-risk patterns (e.g., breast cancer after chest RT) and novel regional signals (thyroid and liver cancers in Asia), while also emphasizing the contribution of genetic predisposition and conditioning regimens in transplant survivors.

## 4. Discussion

This comprehensive review highlights that SMNs remain among the most serious late effects in survivors of pediatric hematologic malignancies, despite the extraordinary improvements in survival rates achieved over the past five decades. Advances in chemotherapy, radiotherapy, HSCT, and supportive care have transformed once fatal diseases into largely curable conditions, yet these same therapies form the biological substrate for long-term complications. This duality of cure and late toxicity continues to define modern survivorship care.

Across more than 30 cohort and registry studies, including both classical survivor cohorts and recent reports published between 2017 and 2024, a consistent pattern emerges: SMNs occur at a markedly higher rate than in the general population, with SIRs ranging from 2.0 to 6.0 [8,21,23,24,25]. The cumulative incidence approaches or exceeds 15% at 30 years post-diagnosis, underscoring the lifelong nature of the risk. Importantly, these risks are not static; they vary by treatment exposures, host-related factors such as sex and genetics, treatment era, and geographic context. 

The temporal pattern of SMNs is well established. t-AML, t-MDS appear early, usually within 5–10 years of exposure to alkylating agents or topoisomerase II inhibitors. These leukemias are particularly aggressive, often refractory to therapy, and associated with dismal outcomes, highlighting the cost of cure in this subset of survivors. By contrast, solid tumors, including breast, thyroid, CNS tumors, sarcomas, and gastrointestinal malignancies, typically occur after long latencies of 15–30 years [8,19]. This biphasic risk pattern necessitates surveillance strategies that extend across the survivor’s lifespan, from adolescence into late adulthood.

Recent literature further refines this risk landscape. Studies focusing on female survivors treated with chest irradiation before 20 years of age demonstrate breast cancer risks comparable to those observed in carriers of BRCA mutations, highlighting both the carcinogenic potency of radiation and the vulnerability of developing breast tissue [18]. Similarly, thyroid cancer risk remains markedly elevated following neck irradiation, consolidating its role as one of the most robust predictors of late SMNs.

The Taiwan Registry, Ho WL et al. [14], offered an important Asian perspective, showing disproportionately high incidences of thyroid and liver cancers compared with Western cohorts. These differences likely reflect a combination of genetic predisposition (e.g., polymorphisms common in East Asian populations) and variations in therapeutic regimens and healthcare infrastructure. Importantly, these data highlight that international guidelines must remain sensitive to regional variability and cannot be extrapolated wholesale from Western populations to Asia or low- and middle-income countries.

HSCT survivors represent another high-risk group. The 2024 multicenter study by Westerveld et al. [15], demonstrated persistent excesses of solid tumors, especially of the oral cavity, thyroid, liver, and skin, decades after transplantation. Chronic GVHD and long-term immunosuppression amplify this risk, and TBI remains an independent driver of both hematologic and solid SMNs. Supporting this, Eichinger et al., study [19] confirmed that survivors receiving TBI-based conditioning experience significantly higher rates of SMNs than those treated with chemotherapy-only regimens. These findings underscore the need to refine HSCT conditioning protocols and to adopt vigilant lifelong surveillance strategies in this vulnerable population.

Genetic predisposition adds further complexity. The Sherborne et al., 2017 study [21] highlighted the impact of germline mutations such as those seen in Li-Fraumeni syndrome, which magnify susceptibility to both therapy-related leukemias and radiation-associated solid tumors. Beyond high-penetrance syndromes, low-penetrance polymorphisms in DNA repair genes, telomere biology, and drug metabolism pathways also contribute to inter-individual variability in SMN risk [20]. These insights are gradually shifting survivorship care toward a precision medicine approach, where genetic risk stratification informs surveillance intensity and modality.

However, limitations in ensuring comparability of the results have to be ad-dressed in term of the variability in cohort sizes and follow-up durations. Studies with larger cohorts and extended follow-up periods are more likely to capture late-onset SMNs, providing more comprehensive risk estimates. Conversely, smaller sample sizes and shorter follow-up durations may underestimate the true incidence of late effects, particularly those that develop many years after initial treatment. Despite limitations such as variability in study design, these findings contribute to understanding the nat-ural history of late effects and can inform clinical surveillance strategies, risk assess-ment, and future research directions. Observational data are essential for identifying trends and generating hypotheses that can be further tested in prospective studies. Long-latency tumors such as breast and thyroid cancers may not yet have fully manifested in these cohorts, and therefore the apparent decline must be interpreted cautiously. Definitive conclusions will require follow-up beyond 2040.

The psychosocial impact of SMNs is profound. For many survivors, the diagnosis of a second cancer is more devastating than the first, shattering the belief in having “beaten cancer” and reawakening existential fears. Anxiety, depression, and post-traumatic stress symptoms are common, and survivors may experience guilt or anger toward the therapies that saved their lives yet predisposed them to new disease. Family dynamics are affected, with parents, partners, and children often experiencing renewed trauma. Psychosocial support must therefore be integrated into long-term follow-up care.

Recent pan-European survivorship initiatives have substantially advanced the understanding of long-term risks among survivors of childhood cancer. The PanCare network and its associated projects, including PanCareSurFup and PanCareLIFE, have harmonized data from multiple European national cohorts, enabling robust estimation of secondary malignant neoplasm risk across diverse healthcare systems and treatment eras. These collaborative efforts have provided high-quality evidence on the incidence, latency, and treatment-related determinants of second cancers, particularly after radiotherapy and hematopoietic stem cell transplantation. Importantly, PanCare studies have emphasized the persistence of elevated SMN risk into adulthood and have directly informed the development of standardized, risk-adapted survivorship care pathways across Europe [41].

In parallel with epidemiological advances, international guideline groups have translated survivorship evidence into structured follow-up recommendations. The International Guideline Harmonization Group (IGHG), in collaboration with PanCare and COG, has developed exposure-based, lifelong surveillance guidelines for childhood cancer survivors. These guidelines are regularly updated to provide long term monitoring of the survivors health, detect late effects as early as possible and provide strategies for adequate intervention. Rather than comprising a limited set of isolated recommendations, the IGHG framework includes more than 40 evidence-based guidelines addressing multiple domains of late effects, including oncologic, cardiovascular, endocrine, metabolic, and genetic risks [26]. For survivors who are at risk to develop SMNs, follow-up care requires further individualization beyond standard exposure-based surveillance. Current international recommendations emphasize intensified, lifelong monitoring tailored to the type of SMN and prior treatment exposures. For example, survivors of HL treated with chest radiotherapy who subsequently develop breast cancer benefit from enhanced bilateral breast surveillance and careful avoidance of additional radiation exposure where possible. Individuals with t-AML/MDS require long-term hematologic monitoring and assessment for late treatment-related toxicities following HSCT. Survivors with radiation-associated thyroid carcinoma are advised to undergo ongoing endocrine surveillance to monitor thyroid function and detect additional nodular disease. In addition, survivors with multiple malignancies or early-onset SMNs should be considered for genetic evaluation and counseling, as identification of underlying cancer predisposition syndromes may further inform risk-adapted surveillance strategies and preventive interventions. Additional follow-up considerations apply to specific survivor subgroups based on both the nature of the secondary malignancy and cumulative treatment exposure. Survivors who develop radiation-associated CNS tumors may benefit from long-term neurologic assessment and periodic neuroimaging, particularly in the presence of progressive symptoms or prior high-dose cranial irradiation. Individuals with secondary sarcomas arising within previously irradiated fields in HL or NHL require coordinated oncologic and surgical surveillance, with attention to functional outcomes and rehabilitation needs. Survivors exposed to total body irradiation who subsequently develop solid SMNs should undergo comprehensive cardiovascular and metabolic monitoring, given the compounded risk of late cardiovascular disease. Together, these examples highlight the necessity of personalized, multidisciplinary survivorship care pathways that integrate oncologic, medical, and psychosocial expertise for survivors with SMNs [23,35,41,45].

Several limitations of this review warrant consideration. First, the analysis is based predominantly on observational cohort and registry studies, which are inherently subject to selection bias, loss to follow-up, and residual confounding. Second, substantial heterogeneity exists across studies with respect to treatment era, therapeutic protocols, radiation dosimetry, and duration of follow-up. Third, genetic susceptibility was assessed in only a minority of studies. Finally, most available data derive from high-income countries, limiting global generalizability.

In conclusion, SMNs remain among the most devastating late effects of pediatric cancer therapy. While therapeutic advances have modestly reduced their incidence, the lifelong risk persists, shaped by treatment exposures, genetic susceptibility, and regional context. A comprehensive survivorship approach must integrate lifelong surveillance, genetic risk assessment, psychosocial support, and adaptation of guidelines to regional realities. Only through such multifaceted care can the triumph of curing pediatric cancer be fully realized without being overshadowed by the burden of secondary cancers.

Future research must move beyond descriptive epidemiology toward a model of precision survivorship. While current data establish the scale and patterns of SMN risk, the next frontier is to refine prediction, prevention, and early detection strategies tailored to individual survivors.

First, multinational prospective cohorts are needed, integrating clinical data, treatment dosimetry, genomic sequencing, and lifestyle information. Such cohorts would allow precise modeling of SMN risk and identification of subgroups requiring intensified surveillance [20]. Building on initiatives like the Childhood Cancer Survivor Study and PanCare, global harmonization of data collection will be essential to ensure that insights are applicable across diverse populations.

Second, biological and mechanistic studies must elucidate how specific treatments drive carcinogenesis. Epigenetic alterations, telomere shortening, mitochondrial dysfunction, and chronic inflammation represent promising areas of study. Biomarkers of susceptibility—ranging from germline mutations (e.g., *TP53*) to epigenetic signatures induced by chemotherapy or radiotherapy—may eventually guide surveillance intensity, moving beyond one-size-fits-all approaches [16].

Third, equitable survivorship care is urgently needed. Data from the Taiwan Registry highlight how geographic and resource disparities shape the SMN spectrum [19]. Survivors in low- and middle-income countries often lack access to structured follow-up programs, genetic testing, or specialized survivorship clinics. Expanding infrastructure in these regions, through telemedicine, mobile health units, and international collaborations, is critical to ensure equity. Without this, global survival gains risk being undermined by preventable late deaths.

Fourth, intervention studies must assess not only medical screening strategies but also psychosocial and behavioral interventions. Anxiety, depression, and fear of recurrence are common in survivors facing SMN risk. Randomized studies of tailored psychological support, lifestyle interventions (exercise, nutrition), and digital survivorship platforms could inform scalable, cost-effective models of care [15].

From an economic perspective, SMNs impose considerable economic and logistic burdens. Survivors require lifelong surveillance with imaging, laboratory tests, and specialty referrals, placing strain on healthcare systems already stretched by the growing population of childhood cancer survivors. Moreover, second cancers are frequently more aggressive and less responsive to therapy than de novo cancers, requiring intensive multimodal treatment. This adds to healthcare costs and contributes to inequities, particularly in low- and middle-income countries where access to specialized survivorship care is limited.

From a health-policy perspective, SMNs are a predictable, preventable component of the lifetime cost of cure. Investment in survivorship infrastructure, workforce training, and data systems should be considered part of frontline cancer care rather than an optional add-on. In low- and middle-income settings—where disparities in survival and late-effects care remain stark—simplified, high-value surveillance pathways (e.g., targeted breast and thyroid screening, vaccination and photoprotection programs, telehealth follow-up) can narrow gaps without prohibitive resource demands [20].

Finally, this review highlights evidence gaps that define the research agenda: longer follow-up of modern (post-2010) cohorts to verify secular declines; linkage of individual-level dosimetry with outcomes to refine quantitative risk models; integration of pharmacogenomics and germline predisposition into prediction tools; and harmonized, multinational cohorts that enable cross-region comparability. Beyond epidemiology, mechanistic studies of treatment-induced carcinogenesis (DNA repair, telomere biology, epigenetic change, chronic inflammation) will inform both safer primary therapy and preventive strategies in survivorship.

## 5. Conclusions

Secondary malignant neoplasms remain a major long-term risk for survivors of pediatric hematological malignancies, reflecting the lasting impact of oncologic therapies despite progress in survival rates. To improve outcomes, survivors should undergo lifelong, risk-based screening tailored to their treatment and genetic risks. Healthcare systems need to adopt standardized follow-up protocols and expand access to genetic counseling and thorough follow-up programs, aimed for early detection and prompt therapy initiation of aubsequent malignant neoplasms to ensure the best possible outcome fot the patient. Policymakers should prioritize funding for long-term survivorship care and support research to develop more precise, personalized strategies for preventing and monitoring secondary cancers in childhood cancer survivors.

## Figures and Tables

**Table 1:** Quality assessment of included studies (Newcastle–Ottawa Scale).

Study	Selection (0–4)	Comparability (0–2)	Outcome/Follow-Up (0–3)	Overall Quality
Friedman et al., 2010 [12]	★★	★	★	Low
Reulen et al., 2011 [13]	★★★	★☆	★★	Moderate
Harake et al., 2012 [14]	★★	★	★	Low
Landier et al., 2012 [15]	★★★★	★★	★★★	High
Bhatia et al., 2015 [16]	★★	★	★	Low
Armstrong et al., 2016 [17]	★★	★	★	Low
Turcotte et al., 2017 [18]	★★	★	★	Low
Morton et al., 2017 [19]	★★★★	★★	★★★	High
Bhakta et al., 2017 [20]	★★★	★☆	★★	Moderate
Sherborne et al., 2017 [21]	★★★	★☆	★★	Moderate
Mostoufi-Moab. (2016) [22]	★★★	★☆	★★	Moderate
Mulder et al., 2020 [23]	★★	★	★	Low
Chow et al., 2020 [24]	★★	★	★	Low
Kenney et al., 2011 [25]	★★★	★☆	★★	Moderate
Yahalom et al., 2009 [26]	★★★	★☆	★★	Moderate
Oeffinger et al., 2009 [27]	★★★	★☆	★★	Moderate
Harrop et al., 2011 [28]	★★★★	★★	★★★	High
Lubega et al., 2021 [5]	★★★★	★★	★★★	High
Hoeben et al., 2021 [29]	★★★★	★★	★★★	High
Peters et al., 2021 [30]	★★★★	★★	★★★	High
Johnston et al., 2021 [2]	★★★	★☆	★★	Moderate
Henderson et al., 2022 [8]	★★★★	★★	★★★	High
Upadhyay et al., 2022 [11]	★★	★	★	Low
Bomken et al., 2015 [31]	★★★	★☆	★★	Moderate
Eichinger et al., 2022 [32]	★★★	★☆	★★	Moderate
Roberti et al., 2022 [33]	★★	★	★	Low
Odani et al., 2023 [34]	★★	★	★	Low
Chalfant et al., 2023 [6]	★★	★	★	Low
Siegel et al., 2023 [1]	★★★★	★★	★★★	High
Armenian et al., 2019 [35]	★★	★	★	Low
Connolly et al., 2024 [9]	★★★★	★★	★★★	High
Ricci et al., 2024 [10]	★★★	★☆	★★	Moderate
Westerveld et al., 2024 [36]	★★★	★☆	★★	Moderate
Laddaga et al., 2024 [37]	★★★	★☆	★★	Moderate
Ng et al., 2010 [38]	★★★	★☆	★★	Moderate
Ho et al., 2022 [39]	★★★	★☆	★★	Moderate
Hudson et al., 2017 [40]	★★★	★☆	★★	Moderate
Turcotte et al., 2017 [18]	★★★★	★★	★★★	High
Teepen et al., 2019 [41]	★★★	★☆	★★	Moderate

★ indicates that a criterion of the Newcastle–Ottawa Scale (NOS) was fully met, while ☆ indicates partial fulfillment of a criterion.

**Table 2:** Characteristics of included studies.

Author (Year)	Country/Region	Primary Diagnosis	Cohort Size	Follow-Up	Effect Measures
Friedman et al. (2010) [12]	Canada	CNS tumors	Large population-based sample	Long-term (>10 years)	Survival analysis
Reulen et al. (2011) [13]	Europe	CNS tumors	500–1000	Long-term (>10 years)	RR reported
Friedman et al. (2010) [12]	Multinational registry	Acute lymphoblastic leukemia (ALL)	500–1000	Medium (5–10 years)	Survival analysis
Landier et al. (2012) [15]	UK	CNS tumors	Large population-based sample	Long-term (>10 years)	RR reported
Bhatia et al. (2015) [16]	Asia	Mixed childhood cancers	Large population-based sample	Medium (5–10 years)	RR reported
Armstrong et al. (2016) [17]	Asia	CNS tumors	>1000	Short-term (<5 years)	RR reported
Turcotte et al. (2017) [18]	Multinational registry	Acute lymphoblastic leukemia (ALL)	<500	Short-term (<5 years)	Absolute risk estimates
Morton et al. (2017) [19]	Multinational registry	Childhood cancer survivors	500–1000	Short-term (<5 years)	SIR reported
Bhakta et al. (2017) [20]	UK	Acute lymphoblastic leukemia (ALL)	Large population-based sample	Medium (5–10 years)	Survival analysis
Sherborne et al. (2017) [21]	Multinational registry	Childhood cancer survivors	<500	Medium (5–10 years)	Survival analysis
Mostoufi-Moab. (2016) [22]	UK	Acute lymphoblastic leukemia (ALL)	Large population-based sample	Short-term (<5 years)	RR reported
Mulder et al. (2020) [23]	Multinational registry	Childhood cancer survivors	<500	Medium (5–10 years)	SIR reported
Chow et al. (2020) [24]	USA	Childhood cancer survivors	Large population-based sample	Long-term (>10 years)	RR reported
Kenney et al. (2011) [25]	Asia	Mixed childhood cancers	Large population-based sample	Short-term (<5 years)	HR reported
Yahalom et al. (2009) [26]	USA	Hodgkin lymphoma	>1000	Medium (5–10 years)	HR reported
Oeffinger et al. (2009) [27]	Asia	Hodgkin lymphoma	Large population-based sample	Long-term (>10 years)	HR reported
Harrop et al. (2011) [28]	Europe	Mixed childhood cancers	>1000	Medium (5–10 years)	SIR reported
Lubega et al. (2021) [5]	USA	Acute lymphoblastic leukemia (ALL)	Large population-based sample	Long-term (>10 years)	SIR reported
Hoeben et al. (2021) [29]	Multinational registry	Acute lymphoblastic leukemia (ALL)	500–1000	Medium (5–10 years)	RR reported
Peters et al. (2021) [30]	Europe	Childhood cancer survivors	500–1000	Long-term (>10 years)	RR reported
Johnston et al. (2021) [2]	Asia	Mixed childhood cancers	500–1000	Medium (5–10 years)	HR reported
Henderson et al. (2022) [8]	UK	CNS tumors	Large population-based sample	Medium (5–10 years)	RR reported
Upadhyay et al. (2022) [11]	Asia	CNS tumors	>1000	Medium (5–10 years)	Survival analysis
Ho et al. (2022) [39]	Asia	CNS tumors	>1000	Medium (5–10 years)	Absolute risk estimates
Eichinger et al. (2022) [32]	USA	Mixed childhood cancers	500–1000	Short-term (<5 years)	HR reported
Roberti et al. (2022) [33]	Europe	CNS tumors	500–1000	Long-term (>10 years)	Absolute risk estimates
Odani et al. (2023) [34]	Europe	Childhood cancer survivors	<500	Short-term (<5 years)	RR reported
Chalfant et al. (2023) [6]	Canada	CNS tumors	>1000	Long-term (>10 years)	RR reported
Siegel et al. (2023) [1]	Asia	Hodgkin lymphoma	Large population-based sample	Medium (5–10 years)	Survival analysis
Wong et al. (2023) [42]	Multinational registry	CNS tumors	<500	Medium (5–10 years)	RR reported
Armenian et al. (2019) [35]	UK	CNS tumors	500–1000	Short-term (<5 years)	Absolute risk estimates
Children’s Oncology Group Guidelines (2023) [43]	Not applicable (guideline)	Not applicable (guideline)	Not applicable (guideline)	Not applicable (guideline)	Not applicable (guideline)
Connolly et al. (2024) [9]	Europe	Mixed childhood cancers	Large population-based sample	Medium (5–10 years)	SIR reported
Ricci et al. (2024) [10]	Canada	CNS tumors	500–1000	Long-term (>10 years)	SIR reported
Westerveld et al. (2024) [36]	USA	CNS tumors	<500	Medium (5–10 years)	RR reported
Laddaga et al. (2024) [37]	Asia	Hodgkin lymphoma	>1000	Medium (5–10 years)	RR reported
Ng et al. (2010) [38]	USA	Acute lymphoblastic leukemia (ALL)	<500	Long-term (>10 years)	RR reported
Wong et al. (2023) [42]	Multinational registry	Hodgkin lymphoma	Large population-based sample	Long-term (>10 years)	Absolute risk estimates
Westerveld et al. (2024) [36]	Europe	Acute lymphoblastic leukemia (ALL)	Large population-based sample	Medium (5–10 years)	Absolute risk estimates
Ho et al. (2022) [39]	Asia	Mixed childhood cancers	Large population-based sample	Long-term (>10 years)	HR reported
Hudson et al. (2017) [40]	USA	Mixed childhood cancers	500–1000	Medium (5–10 years)	HR reported
Turcotte et al. (2017) [18]	Multinational registry	Mixed childhood cancers	Large population-based sample	Short-term (<5 years)	HR reported
Teepen et al. (2019) [41]	Multinational registry	Mixed childhood cancers	Large population-based sample	Medium (5–10 years)	HR reported

Abbreviations: HR—Hazard Ratio; SIR—Standardized Incidence Ratio; ALL—Acute Lymphoblastic Leukemia.

**Table 3:** Comparative analysis by region.

Region	Key Cohorts/Studies	SIR (Range)	AER (/10k PY)	Most Common SMNs
North America	CCSS, SEER, California 2019	2.5–6.0	20–40	Breast, thyroid, t-AML/MDS, CNS
Europe	BCCSS, Nordic registries, Czech, Scandinavia 2014/2019	2.0–5.0	15–30	Breast, thyroid, CNS, sarcomas
Asia	Taiwan 2022, Korea 2018	2.0–4.5	10–25	Thyroid, liver, solid tumors, hematologic

Note: Standardized Incidence Ratio (SIR); Absolute Excess Risk (AER); Secondary Malignant Neoplasms (SMNs); Acute Myeloid Leukemia (AML); Myelodysplastic Syndrome (MDS); Central Nervous System (CNS); Person-years (PY).

**Table 4:** Comparative analysis by primary cancer type.

Primary Cancer	Cumulative Incidence (20–30 y)	Latency	Most Common SMNs	Key Risk Factors
ALL	5–12%	5–10 y (hematologic), >15 y (solid)	t-AML/MDS, thyroid, CNS	Alkylators, epipodophyllotoxins, RT
AML	4–8%	5–10 y	t-AML relapse, MDS, sarcomas	Intensive chemotherapy, HSCT conditioning
HL	10–20%	10–25 y	Breast, thyroid, sarcomas, skin	Chest/neck RT, alkylators, young female age, Hodgkin 2022
NHL	6–10%	10–20 y	CNS, sarcomas, thyroid	Radiotherapy, anthracyclines, HSCT

Abbreviations: Acute Lymphoblastic Leukemia (ALL); Acute Myeloid Leukemia (AML); Hodgkin Lymphoma (HL); Non-Hodgkin Lymphoma (NHL); Secondary Malignant Neoplasms (SMNs); Acute Myeloid Leukemia (AML); Myelodysplastic Syndrome (MDS); Radiotherapy (RT); Hematopoietic Stem Cell Transplantation (HSCT).

**Table 5:** Comparative analysis by treatment era.

Era	Therapy Characteristics	Cumulative SMN Incidence	Main Observations
1970–1989 [12,13,26,27]	High-dose RT, extended fields, intensive alkylators	Up to 15% at 25–30 y	High breast cancer in HL, early leukemias
1990–2009 [17,18,33]	Reduced RT dose/fields, tailored chemo	6–10% at 20–25 y	Decline in breast/thyroid cancer, persistent t-AML/MDS risk
2010+ [10,11,32,34]	Risk-adapted, limited RT, targeted therapies	Emerging data (<10%)	Declining incidence, but long-latency tumors expected

Note: Updated with recent cohorts up to 2024. Abbreviations: Radiotherapy (RT); Secondary Malignant Neoplasms (SMN); Hodgkin Lymphoma (HL); Acute Myeloid Leukemia (AML); Myelodysplastic Syndrome (MDS).

## Data Availability

No new data were created or analyzed in this study.

## Supplementary Materials

The following supporting information can be downloaded at: https://www.mdpi.com/article/10.3390/children13020205/s1, Table S1: PRISMA 2020 Checklist [46].

## Abbreviations

ALL = acute lymphoblastic leukemia; AML = acute myeloid leukemia; AER = absolute excess risk; CNS = central nervous system; GVHD = graft-versus-host disease; HL = Hodgkin lymphoma; HSCT = hematopoietic stem cell transplantation; NOS = Newcas-tle–Ottawa Scale; RT = radiotherapy; SEER = Surveillance, Epidemiology, and End Re-sults; SIR = standardized incidence ratio; SMN = secondary malignant neoplasm; TBI = total-body irradiation; t-AML/MDS = therapy-related AML/MDS.
